# Precision radiotherapy using MR-linac for pancreatic neuroendocrine tumors in MEN1 patients (PRIME): a protocol for a phase I-II trial, and systematic review on available evidence for radiotherapy of pNETs

**DOI:** 10.3389/fendo.2023.994370

**Published:** 2023-05-26

**Authors:** Eline N. M. van Vliembergen, Hidde Eijkelenkamp, Gerlof D. Valk, Menno R. Vriens, Gert J. Meijer, Martijn P. W. Intven, Joanne M. de Laat

**Affiliations:** ^1^ Department of Endocrine Oncology, University Medical Center Utrecht, Utrecht, Netherlands; ^2^ Department of Internal Medicine, Division of Endocrinology, Radboud University Medical Center, Nijmegen, Netherlands; ^3^ Department of Radiotherapy, University Medical Center Utrecht, Utrecht, Netherlands; ^4^ Department of Endocrine Surgical Oncology, University Medical Center Utrecht, Utrecht, Netherlands

**Keywords:** pancreatic neuroendocrine tumor (pNET), multiple endocrine neoplasia type 1 (MEN1), MR-guided radiotherapy (MRgRT), MR-linac, radiosensitivity, radiotherapy

## Abstract

**Background:**

Surgical resection is the standard of care for the treatment of pancreatic neuro-endocrine tumors (pNETs) in patients with Multiple Endocrine Neoplasia Type 1 (MEN1). However, surgery can cause significant short- and long-term morbidity. Magnetic resonance-guided radiotherapy (MRgRT) is a potential effective treatment with little side effects. With traditional radiotherapy techniques, irradiation of pancreatic tumors to high dose levels was hampered by poor visibility of the tumor during treatment. MRgRT uses onboard MRI to guide the treatment, thereby enabling delivery of ablative irradiation doses to the tumor, while sparing surrounding tissues. In this study, we describe results from a systematic review assessing efficacy of radiotherapy in pNET and present the protocol of the PRIME study.

**Methods:**

PubMed, Embase and Cochrane Library were searched for articles assessing efficacy and side effects of radiotherapy for the treatment of pNETs. Risk of bias was assessed using the ROBINS-I Risk of Bias Tool for observational studies. Descriptive statistics were used to describe results of included trials.

**Results:**

Four studies comprising of 33 patients treated by conventional radiotherapy were included. Despite the heterogeneity of studies, radiotherapy appeared to be effective for the treatment of pNETs with most patients responding (45.5%) or stabilizing (42.4%) in tumor size.

**Conclusion and trial design:**

Due to the limited literature available and concerns about damage to surrounding tissue, conventional radiotherapy is currently little used for pNETs. The PRIME study is a phase I-II trial with a single arm prospective cohort study design, investigating the efficacy of MRgRT in MEN1 patients with pNET. MEN1 patients with growing pNETs with a size between 1.0 and 3.0 cm without malignant features are eligible for inclusion. Patients are treated with 40 Gy in 5 fractions on the pNET, using online adaptive MRgRT on a 1.5T MR-linac. The primary endpoint is the change in tumor size at MRI 12 months follow-up. Secondary endpoints include radiotoxicity, quality of life, endocrine and exocrine pancreas function, resection rate, metastatic free and overall survival. When MRgRT is found effective with low radiotoxicity, it could reduce the need for surgery for pNET and preserve quality of life.

**Systematic Review Registration:**

PROSPERO https://clinicaltrials.gov/, (CRD42022325542).

## Background

Pancreatic neuroendocrine tumors (pNETs) are relative rare tumors that can occur both sporadically and hereditary as part of the Multiple Endocrine Neoplasia Type 1 (MEN1) syndrome. Clinical decision making in pNETs is a major challenge. They can present as hormone-producing or non-functional lesions. Hormone producing pNETs are often resected directly upon diagnosis because of the symptoms caused by excessive hormone release by these tumors. Surgical resection is also the standard of care for non-functional pNETs larger than 2.0 cm, due to the increasing risk of metastasis associated with larger lesions (1, 2). Management of pNETs with a size of 1.0-2.0 cm remains controversial because, albeit less frequently than larger tumors, also small tumors have been proven to metastasize (3–5). Surgery, however, causes high morbidity. Major early complications occur in 33% of patients who underwent surgical resection, among which delayed gastric emptying, pancreatic fistula, abscesses, or hemorrhage. In addition, 23% of patients develop long-term complications including new-onset diabetes and exocrine pancreatic insufficiency (6). Further, patients with MEN1 often develop recurrences of pNETs in remaining pancreatic tissue, often requiring reoperation of the pancreas. Current clinical decision making depends on carefully weighing the risk of metastases with the risk of complications of surgery. New treatment methods with lower risk of complications are needed to improve management of early stage pNETs.

Pancreatic NET is one of the pathognomonic tumors of MEN1. Next to the occurrence of pituitary adenomas, and hyperplasia of the parathyroid glands (1). Between 50-70% of MEN1 patients develop pNETs (7–9). Metastatic disease from pNETs is the main cause of MEN1-related death and reduces life expectancy of MEN1 patients (7, 10). A pNET in MEN1 patients is often diagnosed at an early stage, because of screening programs for MEN1-related tumor manifestations. Therefore the national well described cohort of MEN1 patients of the Dutch MEN study group in the Netherlands is an ideal context to evaluate efficacy of new interventions to improve the management of early stage pNETs in MEN1.

Radiotherapy appears to be an alternative local treatment option for pNETs. Previous studies demonstrated that neuroendocrine tumors are radiosensitive (11, 12), but until recently, accurate and precise delivery of high dose radiation therapy to the pancreas has been very challenging. There is a considerable risk of adverse effects because of the proximity of radiosensitive organs such as small bowel loops, duodenum, and stomach. Moreover, the location of the pancreas is dependent on posture and breathing, causing its position to slightly change from moment to moment. pNETs cannot be visualized on contemporary cone-beam CT imaging during irradiation. These technical difficulties are reflected in the current clinical practice where radiotherapy is little used in the management of pNET.

**Figure 1:**
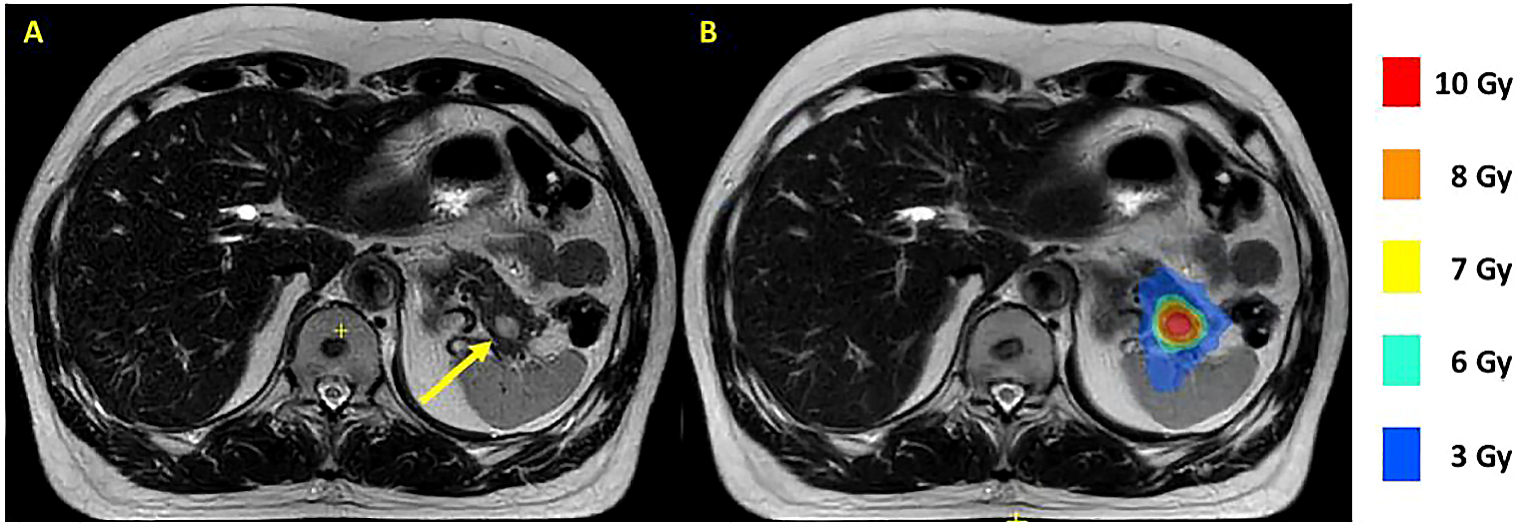
T2 Multivane XD MR scans of a MEN1 patient with a 13 mm tumor in the pancreatic tail made on a 1.5 T MR-linac. The tumor is indicated with a yellow arrow **(A)**. A dose plan with a tumor prescription of 8 Gy is shown with colorwash **(B)**. MEN1, multiple endocrine neoplasia type 1.

Magnetic resonance-guided radiation therapy (MRgRT) holds promise as a new treatment option for pNET. MRgRT enables delivery of ablative irradiation doses to challenging target organs such as the pancreas while limiting the dose to the healthy surrounding tissues (13). MRgRT is delivered with an MR-linac, a combination of a linear accelerator and an MRI scanner. The advantage of using such a system is twofold. First, with the good soft tissue contrast of the MRI, the tumor and surrounding radiosensitive structures are clearly visible during treatment planning and dose delivery, as shown in **Figure 1**. Second, the system enables replanning at each treatment fraction according to the daily on MRI visualized anatomy. These advantages are important when treating pNETs because of the proximity of radiosensitive organs such as small bowel loops, duodenum and stomach (14). MRgRT has recently been studied for the treatment of 16 patients with pancreatic adenocarcinoma (n=13), pancreatic metastasis of renal cell carcinoma (n=2), and metastasis of breast cancer (n=1) (13). Patients were treated with 35 or 40 Gy in 5 fractions. No grade 3 toxicity or higher occurred, showing that use of MRgRT for irradiation of pancreatic tumors is safe. MRgRT is expected to allow treatment of pNETs at an early stage, while causing fewer complications than surgical treatment, preserving quality of life. In addition, a broader patient population could be treated compared to surgical treatment, including patients with unresectable tumors, or patients who are not eligible for surgical treatment due to comorbidities.

In this article we will first discuss the results of a systematic review assessing the current literature regarding effectiveness and toxicity using traditional radiotherapy techniques for the treatment of pNETs. Given the limited availability of data on radiotherapy for pNET, this systematic review was not restricted to MEN1 patients nor early stage pNETs or precision radiotherapy. Second, we present the research protocol of the PRIME study exploring the efficacy of MRgRT for the treatment of asymptomatic pNETs in patients with MEN1.

## Methods

The systemic review was conducted according to the Preferred Reporting Items for Systematic Review and Meta-Analysis (PRISMA) guidelines, using the PRISMA 2020 Checklist (15). The protocol was registered in the PROSPERO international prospective register of systematic reviews (registration number: CRD42022325542).

### Search strategy

The electronic databases PubMed/Medline, Embase and Cochrane Library were searched in March 2023 to review systematically literature published on radiotherapy for pNETs. The literature searches were made with support of a professional librarian from the UMC Utrecht (Appendix 1). Selection of articles was limited to articles published between 2010 and March 2023. There were no language restrictions. The 2010 cut-off point was chosen because of the major developments and progress that radiation delivery techniques have undergone during the last decades.

### Eligibility criteria and study selection

Studies assessing any technique of radiotherapy for the treatment of pNETs in adult patients were eligible for inclusion. Studies with both MEN1 patients and sporadic pNETs were eligible, and with both hormone-producing and non-functional pNETs. Exclusion criteria were treatment of less than three patients and studies only assessing radiotherapy with adjuvant intent, because the effect on the tumor could not be properly assessed when radiotherapy was used as an additional therapy after surgery. Studies using radiotherapy with neoadjuvant intent were eligible if sufficient results were obtained regarding tumor response and toxicity. Based on previous literature and our clinical experience, ​​we were aware that there was only a limited number of studies and patients available for inclusion. For this reason, we did not exclude studies in which other therapies were concomitantly used in addition to radiotherapy (e.g. chemotherapy or somatostatin analogues). Our primary endpoints were the effect of radiotherapy on tumor size and radiation related toxicity.

All identified articles were entered in Rayyan (16), after duplicate removal in EndNote version X9 (Clarivate, Boston, MA) (17). The articles were screened on title and abstracts by two authors (E.v.V. and H.E.) independently. In case of persistent disagreement or doubt, the full text of articles was assessed. Discrepancies were resolved through discussion, with no need of a third reviewer. Of all possible relevant articles full-texts were retrieved, which were again independently screened for meeting the eligibility criteria. Here, disagreements were resolved after discussion with a third author (J.d.L.). Meeting abstracts or conference posters where a full article was not available, were only eligible if sufficient details were reported regarding the number of patients, therapy data, tumor response rate and toxicity. Reasons for exclusion were recorded. Cross-references were checked to search for relevant articles not previously identified in our search.

### Data extraction and analysis

Articles that were eligible for inclusion, were assessed on quality using ROB 2.0 Risk of Bias Tool for randomized controlled trials and ROBINS-I Risk of Bias Tool for observational studies. Data on the study population, pNET characteristics (among which pNET grade according to the WHO classification and tumor grade according to the AJCC grading system), presence of MEN1, radiation modality, irradiation dose and fractionation scheme, type of concurrent other therapy, tumor response rate, toxicity, and the progression-free and overall survival were collected from eligible articles, according to a predefined data-extracting sheet designed by one of the authors. Because of the expected heterogeneity of included studies in terms of population, radiotherapy technique, and concurrent other treatment, narrative data analysis was preferred over statistical analyses.

## Results

### Retrievals and inclusion

**Figure 2:**
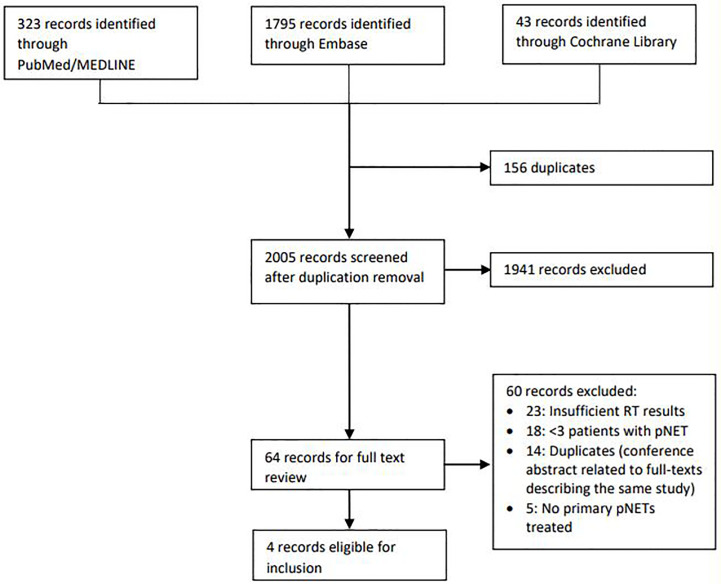
Flow chart illustrating the process of inclusion and exclusion of studies. RT, radiotherapy; pNET, pancreatic neuroendocrine tumor.

Literature search resulted in 2161 records. After systematic removal of duplicates in EndNote, there were 2005 records remaining. After screening on title and abstract, 1941 records were excluded. Frequent reasons for exclusion were conference abstracts with insufficient detail, duplicates that were not automatically detected by reference software, other patient domain (adenocarcinoma) and articles concerning internal radiotherapy (peptide receptor radionuclide therapy). Finally, 64 articles remained for the assessment of the full text. Of these articles, only four were eligible for inclusion. Reasons for exclusion are shown in the flowchart of **Figure 2**. Two of these articles, written by Maidment et al. and Chaudhry et al., were congress abstracts, so no full text articles were available for these studies (18, 19). Furthermore, these studies appeared to have been both conducted in the same center. However, because the abstracts were described in sufficient detail and because the patient characteristics and outcomes in terms of tumor response and toxicity differed between the studies, it was assumed that these were two different studies and it was decided to include both abstracts.

### Risk of bias

There were no low risk of bias studies included. The main biases found were selection bias and reporting bias. With regard to selection bias; in the included studies, mainly patients with high grade pNETs and locally advanced or metastatic disease were included. This can be explained by the fact that radiotherapy was only considered when the standard of care could not be used, e.g. in locally advanced disease, and other treatment modalities were not effective. In addition, very low patient numbers were included. Therefore, the included patients were not a representative sample of the actual patient population. It was unclear what the inclusion and exclusion criteria of participants were and whether the authors included all patients with pNETs who underwent radiotherapy in the mentioned time periods. Only Iwata et al. mentioned to some extent how the patients were included.

With regard to reporting bias; all data collection was undertaken retrospectively. In most studies, some patients received concurrent chemotherapy, which may effected the progression free and overall survival and toxicity outcomes. In the study of Chaudhry et al. it was unclear whether there were patients who received concurrent chemotherapy. In the studies of Chaudhry et al. and Maidment et al., no separate results for the subgroups treated with neoadjuvant, adjuvant or definitive intent were provided. In the studies by Saif et al. and Iwata et al., individualized treatment plan and results were provided enabling to assess results for different subgroups. In all studies follow up was long enough to detect late toxicity and progression-free and overall survival. In conclusion, the included studies were all found to be of low quality.

### Study characteristics

The characteristics of the included studies are shown in **Table 1A**. As expected, there appeared to be heterogeneity between these studies in terms of study design and study population and the studies only included small numbers of patients. Overall, in the four included studies, radiotherapy was used in the treatment of 33 patients with pNETs. Four patients with neuroendocrine carcinomas and three patients treated with neoadjuvant intent were excluded from our analysis. Seven patients with pNETs (21.2%) had no family history with MEN1. In the other studies the presence or absence of a genetic abnormality such as MEN1 was not mentioned. Most patients had non-functional pNETs. The WHO grade was reported for 18 pNETs; all were grade 2 NETs. In most cases there was already locally advanced or metastatic disease before the start of radiotherapy. 28 (84.8%) patients received radiotherapy with definitive intent. 2 (6.1%) patients were irradiated with neoadjuvant and 3 (9.1%) with adjuvant intent. A total of 12 patients (36.4%) received additional systemic therapy after radiotherapy. Seven were treated with capecitabine, 2 with 5-FU and 1 with Teysuno. 2 patients received additional somatostatin analogs.

Table 1APatient and treatment characteristics of included studies.

**Table:** 

	No. patients	No. with MEN1	No. funct. pNETs	No. RT with definitive intent/ no. with surgery	No. receiving chemo-therapy/ no. SSA	Grade of pNETs (WHO)	Tumor stage (AJCC)	Radiation technique	Median RT dose (no. of fractions)
Maidment BW et al., 2012 (19)	11	NR	NR	9/2^c^ (neoadj.)	7/0	NR	3 T3^f^ 8 T4 5 N1 0 M1	NR	50.4 Gy (NR)
Saif MW et al., 2013 (20)	3^a^	NR	NR	3/0	3/1	NR	3 Locally advanced	3DCRT or IMRT	50.4 Gy (28)
Chaudhry H et al., 2013 (18)	12	NR	0	9/3^d^ (adj.)	NR	11 NET-G2 1 NR	1 stage IB 3 stage II 6 stage III 1 stage IV 1 NR	NR	50.4 Gy (NR)
Iwata T et al., 2017 (21)	7^a^	0^b^	2 VIP 1 gastrin	7/0	2/1^e^	7 NET-G2	2 stage III 5 stage IV	7 3DCRT	50 Gy (25)

No, number; MEN1, multiple endocrine neoplasia type 1; funct, functioning; pNET, pancreatic neuroendocrine tumor; SSA, somatostatin analogue; WHO, World Health Organization; AJCC, American Joint Committee on Cancer; RT, radiotherapy; NR, not reported; VIP, vasoactive intestinal peptide; Neoadj, neoadjuvant; Adj, adjuvant; NET-G2, neuroendocrine tumor grade 2; NEC, neuroendocrine cancer; 3DCRT, three dimensional conformal radiation therapy; IMRT, intensity modulated radiotherapy; Gy, gray.

a 3 patients treated with neoadjuvant intent (Saif) and 4 patients with NECs (Iwata) were excluded from our analysis.

b No patient had a family history of pancreatic tumors or MEN. Unclear whether genetic testing has been performed.

c Irradiation of post-resection recurrence was regarded as radiotherapy with definitive intent.

d 3 patients received radiotherapy with adjuvant intent; of 9 patients the intention was not stated, but it was considered that these patients were treated with definitive intent.

e 5 patients received chemotherapy prior to radiotherapy, of whom 2 restarted chemotherapy a few weeks to months after radiotherapy.

f No tumor stages were reported, only TNM-classification was noted.

Table 1BResults of included studies.

**Table:** 

	Radiographic response according to RECIST criteria	Median PFS in months (no. with progression) or PFS rates	Median OS in months (no. died) or OS rates	Acute toxicity according to CTCAE criteria	Late toxicity according to CTCAE criteria
Maidment BW et al., 2012 (19)	2 CR 2 PR 4 SD 3 PD	14.6 (6)	32.1 (5)	1 grade 3 toxicity	1 grade 3 toxicity
Saif MW et al., 2013 (20)	3 PR	1/3 metastases at 13 months	not reached	1/6 grade 1 mucositis^a^ 1/6 grade 2 hand-foot^a^ syndrome	NR
Chaudhry H et al., 2013 (18)	3 CR 3 PR 5 SD 1 PD	1-year = 69% 2-year = 47%	1 year= 89% 2 year= 63%	1 grade 2 nausea and vomiting	1 esophageal stricture 1 mild nausea and early satiety
Iwata T et al., 2017 (21)	2 PR 5 SD	5.5 (5)	55.2 (4)	1 (VIPoma) grade 3 diarrhea 1 (gastrinoma) grade 3 vomiting Vomiting, nausea, diarrhea, mild bone marrow suppression^b^	1 grade 3 gastro-intestinal hemorrhage

RECIST, response evaluation criteria in solid tumors; No, number of patients; PFS, progression free survival; OS, overall survival; CTCAE, common terminology criteria for adverse events; CR, complete response; PR, partial response; SD, stable disease; PD, progressive disease; NR, not reported; VIPoma, vasoactive intestinal peptide producing tumor.

a As toxicity was not reported for the patients individually, it is unknown whether these events occurred in the definitive and/or neoadjuvant intent group.

b Grade of toxicity was not reported, but these symptoms were successfully managed with supportive therapy.

### Tumor response

Overall, in 33 patients, five (15.2%) patients showed complete response, and ten (30.3%) showed partial response. Fourteen (42.4%) patients had stable disease and four (12.1%) had progressive disease. These results are shown in **Table 1B**. In one of two patients with symptoms caused by a functional pNETs there was a reduction of symptoms after radiotherapy.

### Progression-free and overall survival

The progression-free survival and overall survival are also shown in **Table 1B**. Major differences in survival between the various studies were observed, which could be explained by the small numbers of patients, heterogeneity in patients and tumor staging, and the different combinations of concomitant treatments. In Maidment’s study, two of three patients with progressive disease reportedly had distant metastases within less than two months after completing radiotherapy. These metastases might have already been present before start of radiotherapy sessions. Two patients died with no evidence of progressive disease. In Saif’s study, in three patients who received irradiation with definitive intent, one patient developed liver metastases at 13 months of follow-up, none of these patients died. Iwata et al. reported a median progression free survival for patients with pNETs of 5.5 months (95% CI: 3.0 – 28.2 months). Four patients died because of metastasis to other organs or exacerbated hormonal symptoms, at a median overall survival of 55.2 months (95% CI: 4.2 months – not reached). Three of these four patients already had metastatic disease before starting radiotherapy. Chaudry et al. reported only the overall survival rate and recurrence rate, without further information.

### Toxicity

A total of three grade 3 acute toxicities and two grade 3 late toxicities were reported. The degree of late toxicity was unknown for the study by Chaudhry et al, in which one patient had a complicated course with an esophageal stricture and one patient suffered from mild nausea. In the study by Saif et al. it was unknown whether the acute toxicities occurred in the patients who were treated with definitive intent of neoadjuvant intent, as these data were not reported for individual patients; late toxicity was not reported.

## Discussion

This systematic review shows that little research has been done on the treatment of pNETs with radiotherapy. Only four articles published between 2010 and March 2023 met our eligibility criteria. In these studies, 33 patients with pNETs were treated with radiotherapy. The included studies showed that pNETs are sensitive to radiation, as about forty percent had stable disease, one in four patients had partial response and one in eight patients had complete response according to the RECIST criteria. Only twelve percent of the patients had disease progression, despite the fact that most patients included had locally advanced or metastatic disease before radiotherapy.

There appeared to be a lack of high-quality data. In the included studies only small numbers of patients were treated. All studies had a retrospective observational study design, without use of comparison groups. The studies were heterogeneous in terms of patient characteristics (grade of pNETs, tumor stage) and the additional other treatments that were used, which made it impossible to compare the results of the studies. Several patients received concomitant chemotherapy and some patients received somatostatin analogues, which may effected the results. Most participants underwent radiotherapy with definitive intent. However, in some cases radiotherapy was only used with neoadjuvant intent, for initially unresectable locally advanced tumors to become operable. In other patients, radiotherapy was used with adjuvant intent. In the articles by Maidment et al. and Chaudhry et al., no separate results were reported for the patients whose pNETs were irradiated with definitive intent or with (neo)adjuvant intent.

Five grade 3 toxicities were reported in the included studies. However, it was difficult to assess whether this involved radiotherapy-related toxicity alone. The toxicity could also be caused by the concomitant chemotherapy given, or in Iwata’s study, the symptoms could be a direct result of the functional pNETs. In a study of Daamen et al. earlier this year, no acute grade 3 toxicity or higher was reported following radiotherapy for pancreatic adenocarcinoma using MRgRT (13).

Most patients with progressive disease had no growth of the primary tumor, but (further) metastases from a tumor that already was a locally advanced or metastatic stage prior to start of radiotherapy. Therefore, this reported disease progression seemed not attributable to failure of radiotherapy. If pNETs are irradiated at an earlier stage, development of metastases could possibly be prevented.

Our results are consistent with Chan et al. in 2016, to the best of our knowledge the only other systematic review performed on the treatment of radiotherapy for pNETs (12). Besides literature on radiotherapy for the treatment of pNETs, Chan et al. also included studies assessing irradiation of extra-pancreatic neuroendocrine tumors and metastases of neuroendocrine tumors. Two studies (Saif et al. and Maidment et al.) have been included in both our reviews and by Chan’s research group. In line with our systematic review, the included studies were very heterogeneous in terms of population and concomitant other treatment methods. Most studied patients received a high hyperfractionated dose. Acute and late grade 3 toxicity or higher were not comprehensively reported in all studies, but a multitude of complications was reported, such as neutropenia, fatigue, diarrhea, bowel inflammation, sepsis, gastric and duodenal perforation, duodenal stricture and gastrointestinal. Nevertheless, the pooled response rate was 57% (12/21), measured using RECIST or WHO criteria and even more patients had stable disease.

The strengths of our systematic review are the thorough search and independent screening of the appropriate articles by two authors, according to a protocol which was published in Prospero. However, due to the rarity of pNETs and the reluctance to use radiotherapy for the treatment of these neuroendocrine tumors, only a few studies representing a small numbers of participants could be included. Moreover, we also included two conference abstracts, although these abstracts provided sufficient information needed. As mentioned above, the included articles were of low quality and very heterogeneous. This paucity in data from the literature is in accordance to the reluctance physicians have been in the past years with the use of radiotherapy. In the past, radiotherapy has mainly played a role in the treatment of inoperable, locally advanced pNETs, or in the palliation of treatment-resistant pNETs. Despite these limitations in the data, the results of our systematic review demonstrate that pNETs are radiosensitive, supporting the hypothesis that precision MRI guided radiotherapy might fill the unmet needs for less invasive local treatments for pNETs.

At present, treatment options for small non-metastasized pNETs comprise of watchful waiting and surgery. Somatostatin analogs are mainly utilized in advanced and metastasized disease and have shown to slow down the disease process and increase survival. However, there is insufficient evidence for the efficacy of somatostatin analogs in early stage disease as described in the recent study of van Beek et al. (22) Surgery is currently the standard of care for local control in pNETs (1, 23). However, after surgery in 33% of patients early complications (e.g. hemorrhage, pancreatic fistula, delayed gastric emptying) and in 23% late complications (e.g. new onset diabetes mellitus and exocrine pancreatic insufficiency) occur (4, 6, 24). Because of this high morbidity, there is a tendency to watchful waiting in smaller pNETs.

Currently, the local treatment of early stage pNETs is challenging. Although many small pNETs might remain indolent for years, these lesions eventually progress and metastasize (25). Metastatic pNET is a lethal condition and is the primary cause of death in patients with MEN1 (10). Therefore, patients with MEN1 are regularly screened for the presence of pNETs. When pNETs grow to a size larger than 2 centimeters, there is some consensus that the risk of complications from surgery outweighs the risk of complications (1, 2). Unfortunately, in approximately 10% of patients with pNETs smaller than 2.0 cm, liver metastasis do occur before the pancreatic tumor progresses beyond 2.0 cm (3–5). A new treatment modality is needed to achieve local control of early stage pNETs without inducing the extensive morbidity from pancreatic surgery, and this might be achieved with MRgRT. Currently MEN1 patients with pNETs receive annually or semi-annually follow-up scans to monitor the growth of their pNETs. These imaging studies are stressful for patients, as they know each time surgery can be needed if lesions have grown and there is a small risk of metastases. As a result, patients are in suspense for years compromising quality of life (26–28). Moreover, after pancreatic surgery, there is a high risk of recurrence in MEN1 patients in the remaining part of the pancreas, often necessitating subsequent surgery that might again be associated with short- and long term complications. Although surgery reduces the risk of metastases in larger tumors, in as many as 17% of patients, liver metastases are found in long-term follow-up despite the surgery (4).

We hypothesize that high-dose online adaptive MRgRT will fulfill this unmet need. Our literature overview shows that pNETs are radiosensitive, but conventional radiotherapy has not frequently been used because of the proximity of the pancreas to radiosensitive organs such as the small bowel loops and stomach. Moreover, the present literature was not specific for early stage pNETs in MEN1. The MR-linac system can overcome many of the limitations in radiotherapy for pNETs. MRgRT enables delivery of ablative doses of radiotherapy while sparing surrounding tissue by precise tumor localization using 1.5 Tesla MR images just before every radiation therapy session (14). University Medical Center Utrecht has been exploring the safety and technical feasibility of MR-guided radiotherapy in the challenging anatomical area of the pancreas since 2013 (29, 30).

### PRIME study

To explore the efficacy and safety of MRgRT in MEN1 patients with early stage pNET, we designed the PRIME study (https://clinicaltrials.gov/, trial number NCT05037461). The PRIME study is a single-center, phase I-II single-arm and open-label prospective cohort study. The study aims to assess the efficacy and safety of high dose MRgRT using a 1.5T MR-linac for asymptomatic pNET in twenty MEN1 patients with an indication for surgery or smaller growing tumors that will require surgery in the near future if left untreated. Our primary endpoint will be the change in maximal diameter of the pNET at the twelve months follow-up MRI after radiotherapy. Eligible patients will be enrolled from the Dutch MEN Study Group and will be treated at the University Medical Center Utrecht, the Netherlands. The protocol of the PRIME study can be found in Appendix 2. MRgRT is hypothesized to a safe and effective treatment modality for pNETs, which could reduce the need for surgery and may also prevent metastasis, while preserving quality of life.

## Data availability statement

The original contributions presented in the study are included in the article/**Supplementary Material**. Further inquiries can be directed to the corresponding author.

## Author contributions

All authors listed have made a substantial, direct, and intellectual contribution to the work, and approved it for publication.
