# Supplementary material for: Precision radiotherapy using MR-linac for pancreatic neuroendocrine tumors in MEN1 patients (PRIME): a protocol for a phase I-II trial, and systematic review on available evidence for radiotherapy of pNETs
Source: Front Endocrinol (Lausanne). 2023 May 26;14:994370. doi: 10.3389/fendo.2023.994370 (PMC10250693; doi:10.3389/fendo.2023.994370)
Supplement: Supplementary file 1 [file DataSheet_1.docx]

**Appendices**

**Appendix 1**

**Search strategy**

PubMed/MEDLINE

("neuroendocrine tumo*"[Title/Abstract] OR "neuroendocrine neoplas*"[Title/Abstract] OR "neuroendocrine malignan*"[Title/Abstract] OR "neuroendocrine cancer*"[Title/Abstract] OR "neuro-endocrine tumo*"[Title/Abstract] OR "neuro-endocrine neoplas*"[Title/Abstract] OR "neuro-endocrine malignan*"[Title/Abstract] OR "neuro-endocrine cancer*"[Title/Abstract] OR "neuroendocrine Tumors"[MeSH]) AND ("pancrea*" [Title/Abstract] OR "gastroenteropancrea*" [Title/Abstract] OR "duodenopancrea*" [Title/Abstract] OR "duodeno-pancrea*" [Title/Abstract] OR "gastro-entero-pancrea*" [Title/Abstract]) AND ("radiotherap*"[Title/Abstract] OR "radiation*"[Title/Abstract] OR "Radiotherapy"[MeSH Terms])

Embase

('neuroendocrine tumor*':ti,ab,kw OR 'neuroendocrine tumour*':ti,ab,kw OR 'neuroendocrine cancer*':ti,ab,kw OR 'neuroendocrine neoplasm*':ti,ab,kw OR 'neuroendocrine malignan*':ti,ab,kw OR 'neuro endocrine tumor*':ti,ab,kw OR 'neuro endocrine tumour*':ti,ab,kw OR 'neuro endocrine cancer*':ti,ab,kw OR 'neuro endocrine neoplasm*':ti,ab,kw OR 'neuro endocrine malignan*':ti,ab,kw OR 'neuroendocrine tumor'/exp) AND ('pancrea*':ti,ab,kw OR 'gastroenteropancrea*':ti,ab,kw OR 'duodenopancrea*':ti,ab,kw OR 'pancreas’/exp) AND ('radio therap*':ti,ab,kw OR 'radiotherap*':ti,ab,kw OR 'radiation*':ti,ab,kw OR 'radiotherapy'/exp) AND [2010-2023]/py

The Cochrane Library

#1 (neuroendocrine):ti, ab, kw NEXT (tumor* OR tumour* OR cancer* OR neoplas* OR malignan*):ti, ab, kw

#2 MeSH descriptor: [Carcinoma, Neuroendocrine] explode all trees

#3 ("neuro-endocrine"):ti, ab, kw NEXT (tumor* OR tumour* OR cancer* OR neoplas* OR malignan*):ti, ab, kw

#4 ("multiple endocrine"):ti, ab, kw NEXT (neoplas*):ti, ab, kw

#5 #1 OR #2 OR #3 OR #4

#7 (gastroentero OR "gastro-entero" OR duodeno):ti, ab, kw NEXT (pancrea*):ti, ab, kw

#8 (gastroenteropancrea* OR duodenopancrea* OR pancrea*):ti, ab, kw

#9 #7 OR #8

#10 MeSH descriptor: [Radiotherapy] explode all trees

#11 (radio):ti, ab, kw NEXT (therap*):ti, ab, kw

#12 (radiotherap* OR radiation*):ti, ab, kw

#13 #10 OR #11 OR #12

#14 #5 AND #9 AND #13

*additional filter for year of publication (2010-2023) was used

**Appendix 2**

**PRIME Study Protocol**

**General objective**

To assess the efficacy and safety of high dose MRgRT using a 1.5 T MR-linac for asymptomatic pNET in twenty MEN1 patients with an indication for surgery or smaller growing tumors that will require surgery in the near future if left untreated.

**Primary endpoint**

- The change in maximal diameter of the pNET at the follow-up MRI at twelve months after radiotherapy.

**Secondary endpoints**

- Tumor growth or regression or metastasis at long term follow-up (> 12 months);
- Pancreatic surgery;
- Radiation-induced toxicity;
- Health-related quality of life measured by SF-36, EQ5D, and PROMIS 29 profile;
- Signs of endocrine or exocrine pancreatic insufficiency at 12 months, as measured per care protocol by measurement of:
  - Fasting glucose;
  - Blood cell count, serum iron, vitamin B12 and folate;
  - Fecal fat test, and fecal trypsin and elastase.
- Radiologic characteristics of tumor including vascularity;
- Histological characteristics in resected tumors (including fibrosis and tumor necrosis);
- Metastases free survival;
- Overall survival.

**Study design**

The PRIME study is a single-center phase I-II single-arm and open-label prospective cohort study. We plan to enroll twenty patients and treat them at the University Medical Center Utrecht in the Netherlands. A study flowchart of the procedures for participants is illustrated in Figure 1. This trial is registered at https://clinicaltrials.gov/, trial number NCT05037461, and is approved by the institutional Medical Ethics Committee, identification number 21-703.

**Patient selection**

We will enroll patients from the Dutch MEN Study Group (DMSG) prospective cohort. In the Netherlands, people 16 years or older that are diagnosed with MEN1, and treated at one of eight Dutch University Medical Centers, are asked to participate in the DMSG cohort. Participants consent to the collection of their clinical data, filling in questionnaires, and to contact them regarding new studies. Its longitudinal database includes more than 90% of the total Dutch MEN1 population registering screening results, pNET follow-up, and other MEN1 manifestations. Currently, 475 patients are enrolled in the DMSG cohort with data reaching up to 24 years of follow-up for some participants, making it the largest MEN1 cohort in Europe. Therefore, this group is an ideal model for clinical research in pNET.

**Inclusion criteria**

Adult MEN1 patients ($\geq$18 years old) with a pNET measuring between either:

- 2.0 and 3.0 cm;
- 1.0 and 2.0 cm, with moderate growth (2-4 mm/ year) on sequential follow-up scans;
- 1.0 and 2.0 cm and minimal growth (1 mm/ year) reconfirmed on 3 or more sequential follow-up scans;
- 1.0 - 2.0 cm, in situ remaining after previous resection of a larger lesion.

**Exclusion criteria**

- Suspicion of a malignant pNET by the tumor board, including pNET of more than 3.0 cm in diameter or growth of more than 4 mm per year;
- Symptomatic pNET due to hormone production;
- Concurrent somatostatin analog or chemotherapy treatment;
- Peptide receptor radionuclide therapy in the past twelve months;
- History of radiotherapy in the upper abdominal region;
- Contraindication for MRI;
- Pregnancy;
- (Other) metastatic disease, or WHO performance score 3-4.


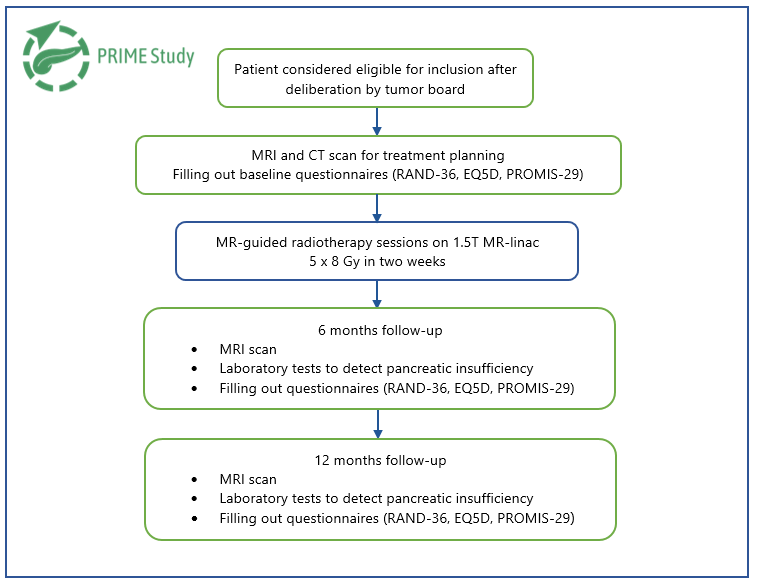


***Figure 1:*** *Flow diagram illustrating the study flow of the PRIME study*

**MR-guided radiotherapy**

Radiotherapy will be delivered on a 1.5T MR-linac using an online adaptive workflow. Patients will receive a hypofractionated scheme of 5 fractions of 8 Gy, every other day in a two week time period. The treatment plan is a 9-14 field intensity modulated radiotherapy (IMRT) plan with dose prescribed to 95% of the PTV.^(31)^

**Response assessment**

The initial response will be assessed at six and twelve months following the start of treatment using 3D T2-weighted MRI. The treatment response will be analyzed based on the RECIST criteria version 1.1. At six and twelve months, we will also collect blood and feces to analyze the pancreatic exocrine and endocrine function. During the study period of twelve months, all follow-up imaging will be performed at UMC Utrecht. If a lesion still measures more than 2.0 cm after radiotherapy or shows growth, an indication for surgery is discussed according to current clinical practice guidelines, and standards of care.

**Safety, toxicity and quality of life**

Patients will be actively monitored for potential radiotoxicity during the study and thereafter through the MEN1 follow-up program of the DMSG at their referring center. Adverse events and radiotoxicity will be defined according to the common terminology criteria of adverse events (CTCAE) version 5. In our experience the risk of radiotoxicity in pancreatic MRgRT is low.^(13)^ However, pancreatic MRgRT is currently used primarily in patients with pancreatic adenocarcinoma who often have a limited life expectancy.

We will assess the QoL by using the SF-36, EQ-5D, and PROMIS-29 questionnaires. Patients will be asked to complete the forms before treatment, and at six and twelve months follow-up. The primary QoL endpoint will be based on the SF-36 score, which measures a generic health status.

**Statistics**

Baseline characteristics will be presented with descriptive statistics. We will analyze the data using a linear mixed model to include covariates such as the baseline tumor size and growth rate. To analyze the QoL, we will use mixed-effects models. Differences in tumor progression and the need for surgical resection will be analyzed by the Kaplan-Meier analysis and the log-rank test. Other continuous outcomes will be analyzed by independent samples t-test and dichotomous outcomes by chi-square. Differences with a P-value <0.05 are considered statistically significant. We will visualize the data using Statistical Package for Social Sciences (SPSS) version 25 (Released 2017. IBM SPSS Statistics for Windows, Version 25.0. Armonk, NY: IBM Corp.).

**Sample size**

The assumption for growth and the impact of radiotherapy on the growth of pNET are based on the studies by Pieterman et al. and by Contessa et al.^(11, 25)^ Pieterman et al. studied the growth rate of 115 pNET lesions in the DMSG database with tumor size <2 cm. In this study, 30% of the lesions showed a 1.6 ± 0.2 mm/ year progression, resulting in an overall growth rate of 0.4 ± 0.2 mm/year. ^(25)^ We will select tumors between 1-2 cm or tumors that have shown progression in follow-up scan; therefore, we consider this estimate conservative. The growth curves show that 58% of this group gets tumor growth when untreated and that their tumor growth rate is relatively stable. Contessa et al. assessed the effects of radiotherapy in 36 patients with progressive pNET at a lower dose compared to our trial. In this study, 39% of patients undergoing radiotherapy had a decrease in tumor size (13% complete response and 26% partial response), and progression stopped in 56% of patients.^(11)^ However, they did not present the exact effects on tumor size. Therefore, we conservatively estimate the effect of radiotherapy at -0.5 ± 0.7 mm/year. Furthermore, we calculated power in case of an effect size of -0.3mm ± 0.5 mm/year, which is the effect size if responders to radiotherapy have only a minimal decrease in size of 1 mm. Based on these assumptions, a minimum of 18 patients must be included in the study to achieve 80% power at a significance level of 5% (two-sided).^(32, 33)^ We expect minimal risk of loss to follow-up because the study is nested in the longitudinal follow-up study of the DMSG, and adherence to follow-up among MEN1 patients is almost complete.
