# Supplementary figures and images for: Precision radiotherapy using MR-linac for pancreatic neuroendocrine tumors in MEN1 patients (PRIME): a protocol for a phase I-II trial, and systematic review on available evidence for radiotherapy of pNETs
Source: Front Endocrinol (Lausanne). 2023 May 26;14:994370. doi: 10.3389/fendo.2023.994370 (PMC10250693; doi:10.3389/fendo.2023.994370)

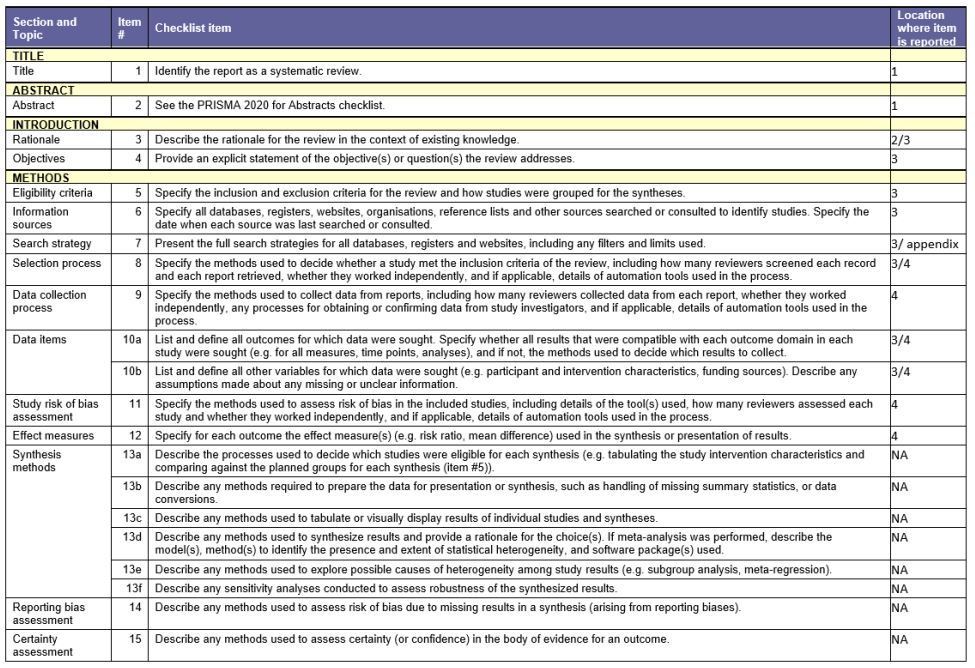

Supplement: Supplementary file 2 [file Image_1.jpeg]

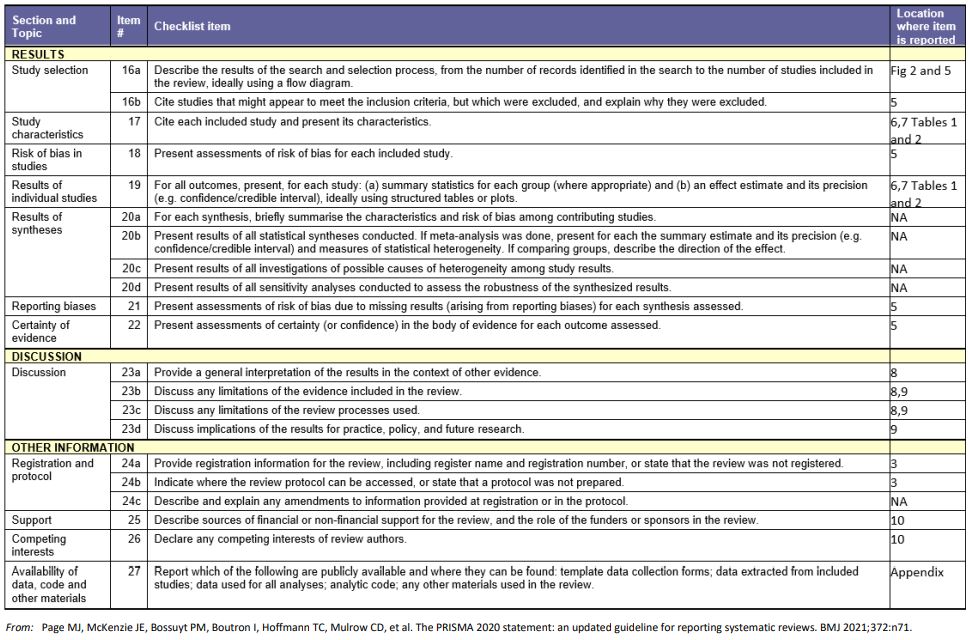

Supplement: Supplementary file 3 [file Image_2.jpeg]
